# Dynamics of Salivary Gland AQP5 under Normal and Pathologic Conditions

**DOI:** 10.3390/ijms21041182

**Published:** 2020-02-11

**Authors:** Kazuo Hosoi, Chenjuan Yao, Takahiro Hasegawa, Hiroshi Yoshimura, Tetsuya Akamatsu

**Affiliations:** 1 Department of Molecular Oral Physiology, Division of Oral Science, Graduate School of Biomedical Sciences, Tokushima University, Tokushima-shi, Tokushima 770-8504, Japan; ychenjuan@tokushima-u.ac.jp (C.Y.); thase@tokushima-u.ac.jp (T.H.); akamatsu_t@tokushima-u.ac.jp (T.A.); 2 Kosei Pharmaceutical Co., Ltd., Osaka-shi, Osaka 540–0039, Japan; 3 Field of Biomolecular Functions and Technology, Division of Bioscience and Bioindustry, Graduate School of Technology, Industrial and Social Sciences, Tokushima University, Tokushima-shi, Tokushima 770-8513, Japan

**Keywords:** aquaporin 5 (AQP5), submandibular gland (SMG), parotid gland (PG), chorda tympani nerve, isoproterenol (IPR), lipopolysaccharide (LPS), mitogen-activated protein kinase (MAPK), nuclear factor-kappa B (NF-κB), phosphorylation

## Abstract

Aquaporin 5 (AQP5) plays an important role in the salivary gland function. The *mRNA* and protein for AQP5 are expressed in the acini from embryonic days E13-16 and E17-18, respectively and for entire postnatal days. Ligation-reopening of main excretory duct induces changes in the AQP5 level which would give an insight for mechanism of regeneration/self-duplication of acinar cells. The AQP5 level in the submandibular gland (SMG) decreases by chorda tympani denervation (CTD) via activation autophagosome, suggesting that its level in the SMG under normal condition is maintained by parasympathetic nerve. Isoproterenol (IPR), a β-adrenergic agonist, raised the levels of membrane AQP5 protein and its *mRNA* in the parotid gland (PG), suggesting coupling of the AQP5 dynamic and amylase secretion-restoration cycle. In the PG, lipopolysaccharide (LPS) is shown to activate mitogen-activated protein kinase (MAPK) and nuclear factor-kappa B (NF-κB) signalings and potentially downregulate AQP5 expression via cross coupling of activator protein-1 (AP-1) and NF-κB. In most species, Ser-156 and Thr-259 of AQP5 are experimentally phosphorylated, which is enhanced by cAMP analogues and forskolin. cAMP-dependent phosphorylation of AQP5 does not seem to be markedly involved in regulation of its intracellular trafficking but seems to play a role in its constitutive expression and lateral diffusion in the cell membrane. Additionally, Ser-156 phosphorylation may be important for cancer development.

## 1. Introduction

Aquaporins (AQPs) are serpentine-type membrane proteins which facilitate water movement across the biological membrane. This water movement is considered to be conducted simply dependent on the osmotic gradient. Since the discovery of the first AQP, AQP1 [1], 13 AQPs, AQP0–12, are presently known to exist in mammals. These AQPs have been divided into 4 major subfamilies; the water-selective AQPs (AQP0, 1, 2, 4, 5, and 6), aquaglyceroporins (AQP3, 7, 9, and 10), superaquaporins, unorthodox AQPs (AQP11 and 12), and AQP8 which have an unusual structure with a long N-terminus, short C-terminus [2]. The general structure of AQPs is as follows: They are 6-transmembrane type proteins having a tandem repeat structure. The molecule has two Asp-Pro-Ala sequences (NPA motif) that form a hemi-channel structure. These structures face each other from inside and outside of the plasma membrane forming a pore through which water can go through. Within the molecule, there are phosphorylation target motifs as well as glycosylation target ones both of them would play pivotal roles in their functions. Today, it is known that AQPs are present in many living organisms; that is, from animals and plants to microorganisms [3]. It is also known that one or several species of AQPs are expressed in individual tissue. Lines of evidence revealed in the last decade have highlighted the diverse function of AQPs beyond water homeostasis [4,5]. Especially data from knock-out experiments, support the involvement of AQPs in brain edema, cell migration, and others, implying that modulation of AQP function or expression could have therapeutic potential [6].

Among various mammalian tissues, kidney convoluted tubule epithelial cells and the salivary gland acinus as well as other exocrine gland tissues transport a large amount of water. In the salivary gland tissues AQP5 is a key water channel like other exocrine glands. Moreover, studies using the *AQP5*-mutant rat [7], *AQP5*-knockout mice [8], and Sjögren’s syndrome [9] and its model mouse of [10] have suggested that AQP5 plays an important role in maintaining the normal physiologyl of the salivary gland. In this review, we focus dynamic changes of AQP5 in the salivary gland under normal and pathologic conditions, in order to understand more about physiology of water transport in the exocrine tissue. This review will elucidate AQP5 transcriptional regulation via neurotransmitter and endotoxin, as well as its posttranscriptional modification.

## 2. Expression and Localization of Salivary Gland Aquaporin 5 during Development, Differentiation, and Regeneration

AQP5 was first identified by the molecular cloning and characterization of a novel *AQP cDNA* from salivary glands as well as lacrimal glands and respiratory tissues [11]. To elucidate the physiological functions of AQP5, its expression and localization in various tissues and cells have been analyzed to date.

AQP5 was first clearly shown to be localized fundamentally at the apical membranes of the secretory acinar cells of the submandibular, parotid, and sublingual glands of rats, and additionally at the apical membrane of the intercalated duct cells of the rat submandibular gland (SMG) [12]. In our previous study, AQP5 is expressed in the apical/lateral and basal membranes of the acinar cells of the rat SMG [7,13].

**Figure 1:**
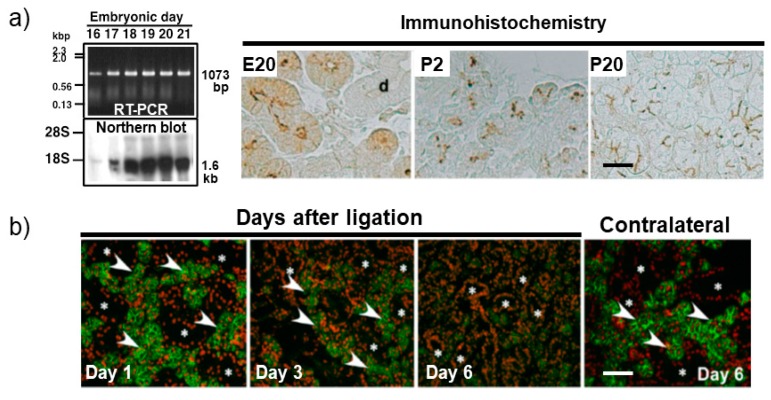
Expression and localization of aquaporin 5 (AQP5) in the developing submandibular gland (SMG) of rats (**a**), and in the ligated SMG of mice (**b**). (**a**) Expression of *AQP5 mRNA* in the embryonic rat SMG was analyzed by reverse transcriptase-polymerase chain reaction (RT-PCR) and Northern blot analyses. Localization of AQP5 protein was analyzed by immunohistochemistry in the rat SMG at the indicated prenatal and postnatal days. In the right panel, “d” indicates typical duct, and a scale bar, 25 µm; (**b**) Effects of duct ligation of the mouse SMG on changes in the immunohistochemical localization of AQP5 was analyzed. Green fluorescence indicates the localization of AQP5 on the indicated day after ligation. Arrowheads and asterisks indicate typical acini and ducts, respectively. A scale bar, 100 µm. (**a**,**b**) modified from [15,27], respectively.

To investigate the physiological role of AQP5 in course of the construction of the salivary function, its expression and localization in the developing salivary glands were also analyzed. King et al. [14] analyzed the expression of AQP5 in the developing rat SMG by means of Western blot analysis and reported its weak expression in the embryonic day E20, and increased expression in the postnatal day P4, P10, and P21. However, they showed only data for four developmental days with no information about the AQP5 localization in these glands. We previously analyzed the expression and localization of AQP5 during development of the rat SMG in more detail [15]. *AQP5 mRNA* was first detected in the embryonic day E16 by means of both reverse transcriptase-polymerase chain reaction (RT-PCR) and Northern blot analyses, and its expression level was dramatically increased by the time of birth (Figure 1a). After birth, its expression is continually detected at all postnatal days analyzed from P0 to P25, but its expression level does not change remarkably. Immunoreactivity for AQP5 in the developing SMG was first observed at embryonic day E18, although *AQP5 mRNA* was expressed at least from the embryonic day E16. It was localized at the considerable area of apical membranes in the terminal portion of the glands, which include proacinar and terminal tubular cells. These AQP5 immunoreactivity seemed to be increase intracellularly and become more evident at embryonic day E20 (Figure 1a). Such intracellular localization of AQP5 at this embryonic day may suggest a vesicular distribution, as described previously [16]. In the course of postnatal development of the rat SMG, the immunoreactivity for AQP5 was observed more intense and was obviously localized at the apical membrane of the submandibular secretory acinar cells in accordance with the differentiation of mature acinar cells (Figure 1a). These observations provide basic data regarding the relation between development of tissue morphology and functional expression of AQP5, in the salivary gland. At the same time, new questions are raised about what is the first signal that initiates AQP5 transcription during development of the salivary gland, which we will address as well. One of the candidate key molecules resolving these questions may be a subtilisin-like proprotein convertase PACE4, because its inhibition and transcriptional silencing suppresses the morphological development and expression of AQP5 in the rat embryonic salivary glands [17]. Additional immunoreactivity for AQP5 was also detected at the intercellular secretory canaliculi of the acinar cells, but not in any duct cells. Larsen et al. [18] reported the expression pattern of AQPs in the developing mouse SMG by means of RT-PCR and Western blotting. *AQP5 mRNA* has been shown to be increased in expression till the birth, which results are similar to our previous data shown in the rat SMG [15]. They detected AQP5 protein at first on the embryonic day E17; its level peaked around birth and at least decreased in the early postnatal day. Aure et al. [19] revealed the expression and localization of AQP5 in the mouse sublingual gland during development. In their study, *AQP5 mRNA* was detected from the embryonic day E13, and its level increased till the early postnatal days by means of real time PCR. On the other hand, AQP5 protein was first detected in the canalicular stage of sublingual gland as a scattered pattern and was clearly organized in the luminal membrane of the acinar cells before birth. During the postnatal development, it was localized in both the luminal and lateral membranes of sublingual acinar cells. Additionally, AQP5 immunoreactivity was observed in the intercalated duct and the entire intralobular duct cells in the terminal bud stage. These developmental data in mice may suggest that AQP5 plays an important role in construction of the salivary functions, although this possibility is still unclear. During development and organogenesis, cell proliferation, migration, invasion, and cell–cell adhesion are frequently occurred. In these processes, AQP1, AQP3, and AQP4 have been suggested to be involved in such cellular processes [20,21,22,23,24]. Therefore, AQP5 may also play an important role in these developmental processes, although it is still unclear.

On the other hand, the salivary gland is known to be a good model of tissue regeneration, that is, the experimental ligation of the main excretory duct of the salivary gland causes the apoptosis of acinar cells and the proliferation of duct cells in both rats and mice [25,26]. Moreover, the reopening of the ligated main excretory duct induces the repopulation and morphological restoration to the normal condition of the gland. In the ligated gland, shrinkage of the acinar cells was remarkably observed together with the decrease in number of acinar cells. Expression level of AQP5 was also decreased by means of Western blotting, whereas, AQP5 was still localized at the apical membranes of the remaining such shrank acinar cells (Figure 1b) in mouse experiments [27]. After reopening of the ligated duct, restoration of the acinar structure was observed depending on the recovery period. The localization of AQP5 was also detected in the apical membrane of the acinar cells in accordance with the restoration of acinar cells, and in intercalated duct cells [28]. Recently, Aure et al. [29,30] showed that the mouse salivary gland homeostasis is regulated predominantly based on the self-duplication of the differentiated acinar cells rather than differentiation of the stem cells under normal and abnormal condition. Shrank acinar cells expressing AQP5 which we observed may be under the pre-stage of self-duplication of acinar cells to restore the salivary gland structure and function. AQP5 may function in these processes, although it is a mere hypothesis at the moment.

## 3. Effects of the Autonomic Nervous System and Chemical Transmitters on Salivary Gland AQP5

The fundamental functions of the salivary glands are (1) to produce the primary saliva in the acinus; (2) transport the saliva through the duct; and (3) discharge the saliva to the oral cavity. In this process, AQP5 is important for production of the primary saliva in the acinus, the secretory end piece. The SMG secretes approximately 60% of total saliva in rodents and humans, while parotid gland (PG) is characterized by secreting large amounts of amylase. The salivary glands are innervated by both sympathetic and parasympathetic nervous systems. A large volume of saliva with low viscosity is secreted by parasympathetic stimulation while small volume with high viscosity one, by sympathetic stimulation. Therefore, it is worth to know whether these autonomic nerves and their chemical transmitters affect the dynamics of AQPs in the salivary glands.

### 3.1. Effects of Chorda Tympani Denervation and Administration of M3 Receptor Agonist on AQP5 in the Submandibular Gland

The parasympathetic nerve which innervates SMG is chorda tympani nerve, the center of which is located in superior salivary nucleus in the medulla oblongata. Li et al. [31] performed denervation of chorda tympani nerve (CTD; parasympathectomy) and examined the level of AQP5 protein in the SMG of rats.

The AQP5, but not AQP1, protein level in the rat SMG was significantly decreased by CTD; the level at four weeks after operation was 37% of that of the un-denervated contralateral gland. In 1 week after CTD, the SMG weight was decreased by 30% which level stayed unchanged until four weeks. Cervical sympathetic trunk denervation (sympathectomy) had little or no effect on the AQP5 and AQP1 protein levels. Administration of cevimeline (an M3 muscarinic receptor agonist), but not pilocarpine, restored the AQP5 protein level reduced by CTD and increased the AQP1 protein level over controls. The *AQP5 mRNA* level was largely unaffected by CTD and cevimeline administration. These results suggest that the AQP5 protein level in the SMG is regulated by the parasympathetic nerves/M3 muscarinic receptor agonist not at transcriptional level but by post-transcriptional mechanism. These authors imply the involvement of lysosomal enzymes in CTD-induced reduction of AQP5 since AQP5 protein level reduced by CTD was increased by administration of chloroquine, a denaturant of lysosomes.

**Figure 2:**
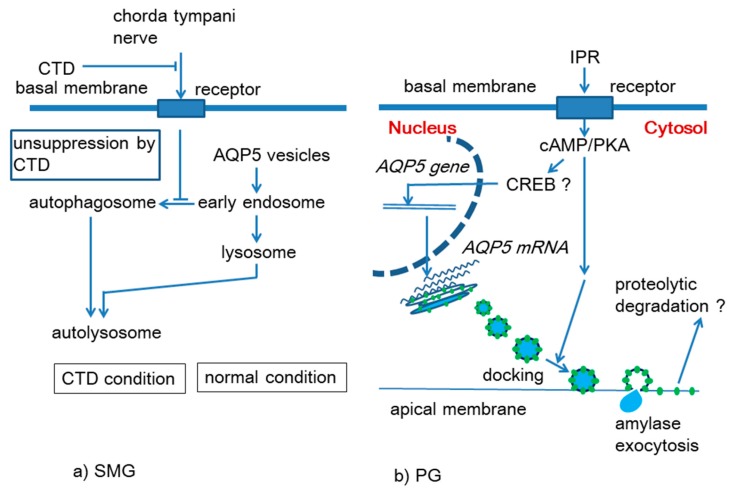
Hypothetical mechanism for regulation of salivary gland AQP5 by the autonomic nervous system and chemical transmitters. (**a**) Suppression of autophagosome activation by chorda tympani nerve leading to maintenance of membrane AQP5 levels in the rat SMG. Chorda tympani denervation (CTD; parasympathectomy) un-suppresses autophagosome activation leading to degradation of AQP5; (**b**) IPR-induced increase in the membrane AQP5 protein and *AQP5 mRNA* in the PG of mice. Green dots in this figure represent AQP5 protein molecule.

Azlina et al. [13] studied effects of CTD on the rat SMG in detail. They reported that lamp2, a lysosomal marker, gradually increased in amount, reaching a peak at the 14th day after CTD. Confocal immunohistochemical analysis disclosed an increased number of lysosome-like structures positive for both AQP5 and lamp2 in SMG acinar cells of the after CTD. Additionally, similar changes were observed for AQP5 and LC3Bs a marker protein of autophagosomes. These data suggest that AQP5 in the SMG entered autophagosomes and/or lysosomes for degradation upon CTD. Through this study these authors performed TdT-mediated dUTP nick end labeling (TUNEL) assay and confirmed that no increase in the number of apoptotic cells in the SMG by CTD. Small reduction in the gland weigh [13,31] seemed to be due to autophagy since the expression of LC3B-II, a marker protein of autophagosomes was transiently (at 1–3 days) induced [13]. These studies suggest that the AQP5 level in the SMG is maintained by the chorda tympani nerve, which is constantly sending a basal level of neural signal to the gland [32,33]. Obstruction of such neural signal would cause the elevation in the number of autophagosomes and/or in lysosome activity, resulting in reduction of the AQP5 protein level. Hypothetical mechanism representing this idea is shown in Figure 2a.

### 3.2. Effects of Isoproterenol on AQP5 in the Mouse Parotid Gland

Mouse PG acinar cells are filled with abundant secretory granules containing amylase. They are exocytosed by administration of β-adrenergic receptor agonists via increased intracellular cAMP following adenyl cyclase activation [34,35]. On the other hand, β-adrenergic agonists and their second messenger, that is, cAMP and its derivatives, are also known to increase expressions of the *AQP5 mRNA* and protein in MLE-12, a cultured mouse lung cell line, and induce translocation of AQP5 to the apical plasma membrane [36]. Thus Chen et al. [37] examined the possibility that β-adrenergic agonists affect AQP5 in the salivary gland; that is, they studied the effects of IPR, a β-adrenergic agonist, on AQP5 in the mouse PG.

In the membrane fraction of mouse PG, the AQP5 protein level was increased by IPR injection (*ip*) at 1 h, and remained high up to 6 h; this change coincided with the secretion of amylase. Thereafter, AQP5 level decreased and returned to their original level at 12–48 h. After IPR injection, the level of *AQP5 mRNA* increased gradually and reached a maximum at 24 h. The facts suggest a rapid appearance of AQP5 at plasma membrane by IPR, and such appearance is believed to be the result of exocytotic translocation of AQP5 from the granule membrane to the plasma membrane. In fact, the membrane of PG secretory granules is reported to bear the AQP5 protein [38]. Their results also indicate that IPR induces *AQP5 gene* transcription to produce its *mRNA*. This hypothesis is presented in Figure 2b.

These authors imply that reduction of AQP5 starting 6 h after IPR injection would be due to proteolysis of AQP5. From both in vivo and in vitro experiments using various inhibitors the responsive enzyme for this proteolysis is suggested to be calpain [37]. Despite β-adrenergic agonist was shown to elevate the *AQP5 mRNA* level in the PG, it is still uncertain how *AQP5* transcription is regulated by cAMP in this tissue.

## 4. Downregulation of Salivary Gland AQP5 by Lipopolysaccharide via Cross-Coupling of Transcription Factors

In addition to secretion of water, the salivary glands secrete also electrolytes, various proteins, glycoproteins, and mucins to aid in digestion, cleansing, and protection of the oral cavity. There is a possibility that AQP expression is influenced under clinical condition, like sialolithiasis and chronic sialadenitis. Lipopolysaccharide (LPS) is one of the best studied immunostimulatory components of bacteria and can induce systemic inflammation and sepsis [39]. LPS is the major component of the outer envelope of Gram-negative bacteria, which is thought to be a cause of the widespread cellular activation observed in patients suffering from Gram-negative septic shock. LPS-induced activation of pro-inflammatory cytokines and anti-peptides is conducted through a membrane receptor, Toll-like receptor 4 (TLR4), and induce innate immune responses. Upon recognition of LPS by the extracellular domain of TLR4, TLR4 undergoes oligomerization and links to its downstream adaptors through interactions with the domains of TIR (Toll-interleukin-1 receptor), ultimately resulting in activation of the transcription factor nuclear factor-kappa B (NF-κB) and increases in transcription of various pro-inflammatory cytokines. In addition to the NF-κB pathway, the mitogen-activated protein kinase (MAPK) pathway is also activated. Activation of downstream MAPK pathways leads to the induction of the growth factor-/chemical inducer-activated ERK1/2 pathway and the cytokine-inducible JNK and p38 pathways.

It has been reported that AQP4 protein levels in human primary cultured cortical astrocytes is decreased by p38 and JNK inhibitors [40], and that AQP3, -5, and -8 expression induced by hyperosmotic stress in cultured astrocyte is suppressed by ERK inhibitors [41]. These data imply that MAPK pathway regulate some of AQP protein levels. Additionally, p38 MAPK in intrinsic renal cells and infiltrating leukocytes is reported to be correlated with renal dysfunction and histopathology in human glomerulonephritis [42].

IL-1β, one of the inflammation cytokines, is induced and secreted into the saliva by LPS stimulation in mice [43,44]. On the other hand, pilocarpine-stimulated saliva production is reduced by more than 50% in the LPS-injected C3H/HeN male mice. The transcription levels of salivary gland *AQP5* and *AQP1 genes* were decreased by LPS in vivo, and the mechanism for signal pathways underlying this decrease were investigated by in vitro experiment using mice [45]. In an organ culture system, the LPS-induced transcriptional inhibition, that is, reduction of the *AQP5 mRNA* level by LPS, is blocked completely by pyrrolidine dithiocarbamate (PDTC) and MG132, which are inhibitors for IκB kinase and 26S proteasome, respectively. Similarly, MAPK pathways inhibitors, AG126 and SP600125 (inhibitors for ERK1/2 and JNK, respectively) strongly suppressed the inhibition of *AQP5* transcription by LPS. These results clearly demonstrate that not only NF-κB but also MAPK pathways are involved in the transcriptional inhibition of *AQP5* by LPS. In contrast, AQP1 downregulation was not blocked by inhibitors of NF-κB and MAPK pathways, probably because no NF-κB-binding element was present in the *AQP1* promoter. By this experiment, the downregulation effect of LPS on AQP5 is completely blocked by either one of the inhibitors for NF-κB, p-c-Jun, or c-Fos pathway, indicating that all 3 of these transcription factors are indispensable for this mechanism. Yao et al. [45] hypothesized that p65, p-c-Jun, and c-Fos form a ternary complex, which complex then binds to *AQP5* promoter. To confirm this possibility co-immunoprecipitation experiments were performed. PG nuclear extracts from mice 3 h after LPS injection and those from non-treated mice were incubated with each Sepharose columns which had been coupled with either anti-p65, anti-p-c-Jun, or anti-c-Fos antibodies. The eluates from these antibody-Sepharose columns were subjected to Western blot analysis and probed with anti-p-c-Jun antibody. In the eluates from anti-p-c-Jun and anti-c-Fos antibody Sepharose columns incubated with non-treated mouse PG nuclear extract, a small amount of p-c-Jun was detected. However, no detectable amount p-c-Jun was present in the eluate from the anti-p65 antibody Sepharose column. On the other hand, the same analysis conducted for PG nuclear extract obtained from LPS-injected mice showed that p-c-Jun was strongly detected in the eluates from not only anti-p-c-Jun and anti-c-Fos antibody Sepharose columns but also anti-p65 antibody Sepharose column. These results suggest that in the PG of normal mice, the p-c-Jun/c-Fos complex is constitutively formed. This complex however does not suppress *AQP5* transcription since it is not binding p65. Upon LPS stimulation, on the other hand, the level of 3 transcription factors (p65, p-c-Jun, and c-Fos) are elevated; they form a complex, which results in suppression of *AQP5* transcription by binding to *AQP5* promoter.

**Figure 3:**
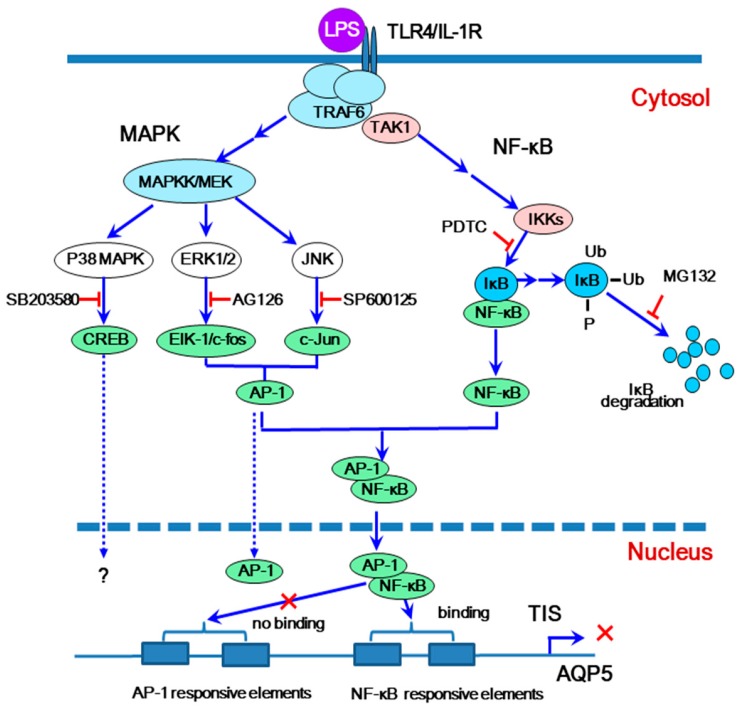
Hypothetical mechanism for lipopolysaccharide (LPS)-induced signaling and downregulation of *AQP5* transcription via cross-coupling of mitogen-activated protein kinase (AP-1) and nuclear factor-kappa B (NF-κB) (a model proposed from mouse experiment). TIS, transcription initiation site.

Two NF-κB and 2 AP-1 responsive elements, though they are putative, are found in the promoter of the mouse *AQP5 gene*. To evaluate possible changes in the *DNA*-binding activity of nuclear extracts to these responsive elements, electrophoretic mobility shift assay (EMSA) was performed by using 2 NF-κB binding probes and AP-1 binding probes. The results indicate that the binding activity of nuclear extracts to the *DNA* probe corresponding to the two NF-κB-responsive elements of the *AQP5* promoter was increased after LPS stimulation. However, the *DNA*-binding activity of nuclear extract toward the two AP-1-responsive elements was not raised after LPS injection. The EMSA results suggest that the complex of p65/p-c-Jun/c-Fos induced by LPS directly binds to the 2 NF-κB-responsive elements but not to the AP-1-responsive elements. Moreover, the nuclear translocation of p65 and p50 (the subunits NF-κB) triggered by LPS is inhibited by PDTC and MG132, which are an inhibitor for I-κB kinase and proteasome (inhibitor for degradation of ubiquitin-conjugated proteins also), respectively. Taken together, the above data confirms that the transcription factors, p65, p-c-Jun, and c-Fos are activated by NF-κB and MAPK pathways and that all 3 factors make a ternary complex which is necessary for downregulation of *AQP5* transcription. It is also confirmed that *DNA* binding of the nuclear extract to NF-κB responsive elements, but not to AP-1 responsive elements is elevated by LPS injection. The hypothesis for this mechanism is schematically shown in Figure 3.

Schüle et al. co-transfected rat osteoblast ROS17/2.8 cells with Jun and Fos expression vectors and made these cells express Jun/Fos proteins. In these cells, they reported that transcription of the *osteocalcin gene*, which has a consensus sequence of AP-1 response element, was suppressed under not only vitamin D_3_-stimulated conditions but also un-stimulated conditions [46]. These authors termed such unexpected interactions between distinct transcription factors as cross-coupling [47]. Stein et al. found that the bZIP region of c-Fos and c-Jun enabled to interact physically with NF-κB p65 and that the complex of p65/Jun or p65/Fos exhibited enhanced *DNA* binding and biological function [48]. In activated T cells, AP-1-NF-κB ternary complex formation was also visualized [49]. Our opinion in this section is that the NF-κB transcription factor is directly involved in the AQP5 downregulation in the LPS-stimulated PG by cross-coupling with p-c-Jun/c-Fos transcription factors.

## 5. Post-Translational Modifications of AQP5

Reversible post-translational modifications, such as phosphorylation and ubiquitination, play roles in regulating the expression level as well as channel activity of AQPs on the cell membrane. The membrane AQP abundance is usually regulated by intracellular trafficking between cytoplasmic vesicles and cell membrane, that is, exocytosis and endocytosis. Phosphorylation is involved in exocytosis of some AQPs, such as those seen in mammalian AQP1, AQP2, and AQP4 [50,51,52]. In addition, ubiquitination is known as an endocytosis signal in AQP2 [53]. The transport activity of water and small molecules through a pore in the AQP molecule, that is, channel gating, can be regulated by structural changes of the channel molecule. Phosphorylation-related channel gating is well known in plant AQPs; its existence is also suggested in yeast AQPs and mammalian AQP0 [54,55,56]. For better understanding of the intracellular regulations of AQP5 by post-translational modifications, we, in this section, review current knowledge about the phosphorylation and ubiquitination of AQP5.

At first, we describe the post-translational modifications of mammalian AQP2 because AQP5 protein share high sequence similarity with AQP2 protein and both AQPs are often compared. Mammalian AQP2, a vasopressin-regulated AQP, is a well-characterized AQP concerning the roles of phosphorylation and ubiquitination in the intracellular trafficking. Four phosphorylation sites of human AQP2 have been identified; these are at Ser-256, Ser-261, Ser-264, and Thr-269 (Ser-269 in mice). Phosphorylation at each of these sites is conducted by a vasopressin-dependent manner; that is, vasopressin stimulation increased phosphorylation of Ser-256, Ser-264, and Thr-269, and decreased that of Ser-261 [57,58,59,60]. Among of them, phosphorylation at Ser-256 is induced rapidly by vasopressin-regulated cAMP signaling and plays critical role in the exocytosis of AQP2 [52]. On the other hand, AQP2 is ubiquitinated at Lys-270 (short-chain ubiquitination) by vasopressin removal or protein kinase C activation, and this modification plays a role in enhancement of the endocytosis and lysosomal degradation of AQP2 [53]. Thus, membrane AQP2 abundance regulated by exocytosis and endocytosis are controlled by a cAMP-dependent phosphorylation and ubiquitination, respectively.

### 5.1. Phosphorylation Sites of AQP5 and Related Signals

In AQP5 phosphorylation, a cAMP signaling pathway mediated by protein kinase A (PKA) is of particular interest. AQP5 phosphorylation was first detected in mouse alveolar MLE-12 cells after long-term exposure (8 h) to cAMP [36]. These authors also reported that AQP5 phosphorylation was detected in AQP5-proteoliposomes, which had been incubated with PKA but not with protein kinase C or casein kinase II, although they failed to show actual phosphorylation site [36]. In AQP5 of most species, 2 consensus motifs are conserved as PKA-target phosphorylation sites: One is RRTS-156 located at an intracellular loop D; the other is RKKT-259 located at C-terminal region, which is corresponding to the motif including Ser-256 of AQP2. Both Ser-156 and Thr-259 of AQP5 of distinct species were experimentally clarified as the phosphorylation sites. Human AQP5 expressed in human bronchial epithelium BEAS-2B cells is phosphorylated at its Ser-156, and mouse AQP5 expressed in human salivary gland HSG cells is phosphorylated at its Thr-259 [61,62]. Phosphorylation of each site is enhanced by cAMP analogues and by cAMP increasing agents such as forskolin [61,62]. Other phosphorylation sites and related kinases targeting them remain unidentified.

### 5.2. Roles of cAMP-Dependent AQP5 Phosphorylation

Unlike AQP2, cAMP-dependent phosphorylation of AQP5 does not seem to be markedly involved in regulation of intracellular trafficking of AQP5. Replacement of Ser-156 (S156A) or Thr-259 (T259A) of AQP5 with non-phosphorylated alanine by in vitro mutagenesis had no apparent effect on AQP5 membrane localization comparing to wild-type AQP5, even upon stimulation of cAMP signaling [61,62,63,64,65]. On the other hand, the level of membrane expression of a phosphomimetic S156E mutant AQP5 was higher than that of wild-type one and S156A mutant under a non-stimulated condition, suggesting that the phosphorylation at Ser-156 is involved in constitutive membrane expression [64]. Recently, Biswas et al. also reported that the membrane expression and stability of AQP5 may be regulated by the phosphorylation at Ser-156 [66]. In addition, time-lapse imaging of quantum dot-labelled surface AQP5 suggested that lateral diffusion of AQP5 within cell membrane is regulated by Thr-259 phosphorylation [65]. Taken together, cAMP-dependent phosphorylation of AQP5 may play a role in its constitutive expression and lateral diffusion in the cell membrane.

Phosphorylation of AQP5 at Ser-156 also seems to be involved in several processes of cell growth. Expression of wild-type AQP5 but not that of S156A mutant in culture cells induced cell proliferation by activating Ras signaling pathway and cell differentiation, migration, and invasion, by mediating c-Src signaling pathway [67,68]. In a mouse xenograft model, transplanted cells expressing wild type AQP5 markedly formed tumor nodule, whereas transplanted cells expressing S156A mutant did not [69]. In fact, AQP5 is expressed in cancer cells of many kinds of tissues such as the lung, breast, liver, stomach, and colon [67,70,71,72,73]. In addition, phosphorylated AQP5 was detected in the lung cancer tissues [69]. These observations indicate that cell proliferation, differentiation, migration, and invasion, all processes of which are leading to the tumor progression, are regulated by AQP5 expressed in a manner of the phosphorylation of AQP5 at Ser-156.

The cAMP-dependent phosphorylation of AQP5 is involved also in physiological exocrine event. AQP5 phosphorylation was detected in lacrimal gland administrated with eye drop containing pituitary adenylate cyclase-activating polypeptide, suggesting that this phosphorylation participates in tear secretion [74]. In salivary glands of mice injected with IPR, phosphorylated AQP5 at Thr-259 was rapidly and transiently detected, and was localized at luminal plasma membrane, suggesting that this phosphorylation participates in a process of salivary protein secretion [61]. Thus, in vivo induction of AQP5 phosphorylation by the receptor-mediated cAMP signaling in non-neoplastic cells of normal organ shows physiological importance of this phosphorylation.

### 5.3. Possibility for Phosphorylation-Regulated AQP5 Gating

Phosphorylation-regulated transport activity is well known in plant AQPs. In spinach SoPIP2;1, for example, phosphorylation at Ser-115 in the cytosolic loop B or at Ser-274 in the C-terminal region increases water transport activity probably by opening the lid which is formed by loop D near the water pore in cytoplasmic side [54,75].

In AQP5, the phosphorylation-dependent gating remains unknown but there is this possibility. In analyses by using the budding yeast, water and H_2_O_2_ permeability through rat AQP5 was transiently enhanced at pH 7.4 by cAMP produced following glucose-induced way, without increasing membrane expression of AQP5. This observation implies that AQP5 is possibly gated in a phosphorylation-dependent manner [76]. Further functional evidence is required to clear the possibility and mechanisms of phosphorylation-dependent gating of AQP5.

### 5.4. Ubiquitination of AQP5

In contrast to AQP5 phosphorylation, AQP5 ubiquitination was described only in a few reports. Without experimental data, AQP5 ubiquitination was first described in the article concerning AQP2 ubiquitination [53]. Next, AQP5 ubiquitination was slightly detected in mouse SMG [77]. Thus, concerning AQP5 ubiquitination, almost all issues such as modification sites, related signals, and roles remain unresolved.

## 6. Conclusions

AQP5 protein is expressed in the acinus as early as embryonic day E17–18 and it is continued to be expressed afterward. Downregulation of SMG AQP5 by denervation of the parasympathetic nerve is shown to be due to activation of autophagosomes. However activation mechanism of AQP5 synthesis after such downregulation is unknown. Similarly, mechanism for IPR-induced upregulation of *AQP5* transcription in the PG is not determined as well. These issues would be important for better understanding AQP5 function in the salivary glands. Mechanism for LPS-induced downregulation of AQP5 in the PG is well described. Physiological meaning of effects of LPS, however, still needs to be elucidated. AQP5 has at least two phosphorylation sites in the molecule. Phosphorylation of these sites seems to play a role in its constitutive expression and lateral diffusion in the cell membrane. AQP5 also may be involved in cell proliferation, differentiation, migration, and invasion of tumor. These important issues need to be clarified.

## Abbreviations

AP-1	activator protein-1
AQP	aquaporin
CTD	chorda tympani denervation
IPR	isoproterenol
LPS	lipopolysaccharide
MAPK	mitogen-activated protein kinase
NF-κB	nuclear factor-kappa B
PDTC	pyrrolidine dithiocarbamate
PG	parotid gland
PKA	protein kinase A
RT-PCR	reverse transcriptase-polymerase chain reaction
SMG	submandibular gland
TLR4	Toll-like receptor 4
TUNEL	TdT-mediated dUTP nick end labeling
